# The Alexander von Humboldt-Foundation

**DOI:** 10.1186/1878-5085-5-S1-A22

**Published:** 2014-02-11

**Authors:** Alexander von Humboldt-Stiftung

**Affiliations:** 1 Jean-Paul-Str. 12, 53173 Bonn, Germany

## 

The Alexander von Humboldt Foundation promotes academic cooperation between excellent scientists and scholars from abroad and from Germany. To this end, it grants more than 700 research fellowships and research awards annually. These allow scientists and scholars from all over the world to come to Germany to work on a research project they have chosen themselves together with a host and collaborative partner. Scientists or scholars from Germany can also profit from the support and carry out a research project abroad as a guest of one of well over 26,000 Humboldt Foundation alumni worldwide – the Humboldtians.

Once a Humboldtian, always a Humboldtian. Even after the stay in Germany has come to an end, the Humboldt Foundation maintains close links with their alumni. The alumni sponsorship is tailored to the needs of every single Humboldtian, providing flexible support for the particular development and path in life as well as for cooperation with others. The Humboldt Network includes 49 academics who have been awarded the Nobel Prize. In 2011, the Nobel Prize for Medicine was divided among Humboldtians Bruce A. Beutler from the United States, Jules Alphonse Hoffmann from Luxembourg and Canadian Ralph M. Steinman. Beutler and Hoffman jointly received one half of the prize for their discovery of innate immunity. Steinman received the other half for his discovery of the dendritic cell and its role in adaptive immunity.

From 2008 to 2012, a total of 2,830 fellowships were granted to academics abroad from all disciplines. For the same period, the Humboldt Foundation received 1,779 applications and approved 526 fellowships to researchers from the life sciences. The application and approval figures for medicine for the years 2008 to 2012 were 260 and 63, respectively.

As an intermediary organisation for German foreign cultural and educational policy the Humboldt Foundation promotes international cultural dialogue and academic exchange. The Alexander von Humboldt Foundation is funded by the Federal Foreign Office, the Federal Ministry of Education and Research, the Federal Ministry for Economic Cooperation and Development, the Federal Ministry for the Environment, Nature Conservation and Nuclear Safety as well as other national and international partners.

## More information

http://www.humboldt-foundation.de

